# What They Did

**Published:** 1872-04

**Authors:** 


### WHAT THEY DID.

SOME men have occupied a large space in their day and generation, but after working hard and living long, all that they really accomplished can be told in a line. Multitudes who made a stir in their time can have their history, their life work, told in a sentence, in a word; and of other multitudes the historian feels it necessary to record only the year of their birth and death. One of the most suggestive books, in this direction, ever prepared, is Hole's "Brief Biographical Dictionary," published by Hurd and Houghton some years ago, giving one line to every eminent name, what they did, when born and when they died; now and then, in very remarkable cases, two or more lines.

"Henry Clay, American statesman, b. 1777, d. 1852.

"Daniel Webster, American jurist and statesman, b. 1782, d. 1852.

"McDonald Clark, American poet, b. 1798, d. 1840."

Of many of the great, of many who attracted a large share of public attention while acting on the stage, only a few things are remembered of what they have done, or their striking sayings; and one by one even these fall off and pass out of notice, until at length a single saying only remains to carry our memories back to the fact that they ever existed.

"Be sure you're right, then go ahead!" said Davy Crockett; and only by this short sentence does he live at all. "I'd rather be right, than President!" is the reported exclamation of Henry Clay, the great commoner, the greatest orator of his generation.

"I still live!" three short words, not a dozen letters in all, are those in which we are reminded oftenest of "the god-like."

"The Union, it must and shall be preserved!" is the remnant of all that Andrew Jackson said and did, which oftenest carries us back to his name.

Reader, what act done, what sentence recorded, shall make you immortal among men?

There are others again who seem to have been born, to have lived and struggled, for the express purpose of doing one thing, and that at the time considered as a trifling episode of life, or a mere accident. Heber has left only one memento of a short but busy life, and that the result of a happy thought of a friend while he was on a visit to his father-in-law; the inspiration which produced the hymn "From Greenland's icy mountains."

The glorious and immortal Isaac Watts called upon a lady friend one day, to chat awhile. Night came on, and he was pressed to stay until the morning, and then until tea again, and then not to go at all; and there he remained twenty years, and wrote the hymns which have handed his name down to the ages.

The "Marseilles Hymn" is all that is left of the doings of its author's long life; while the whole Christian world knows no more of what Miss Elliott did, than the hymn, "Nearer my God to Thee!"

At the same time no man or mote is made in vain; each has had a niche prepared for him, and to be filled by him, in the designs of the Omnipotent One, who never made a mote in vain; yes, a niche to be filled, as a brick larger or smaller in the great building of Almighty design, to carry out his great purposes. Those who willingly and lovingly fill the space intended for them, are the children of the Mighty One; those who are made to perform their part against their wills, with no wish to aid the work, but are used in spite of them, to such are reserved the "blackness of darkness" of the pit.

